# The African cancer advocacy consortium: shaping the path for advocacy in Africa

**DOI:** 10.1186/1750-9378-8-S1-S8

**Published:** 2013-07-15

**Authors:** Folakemi T Odedina, Kwanele Asante-Shongwe, Emmanuel J Kandusi, Richard Segal, Shannon Pressey, R Renee Reams, Virgil H Simons

**Affiliations:** 1 Pharmaceutical Outcomes and Policy, College of Pharmacy, University of Florida, Gainesville, FL, USA; 2 BreastSens, Johannesburg, South Africa; 3 Tanzania 50 Plus Campaign, Tanzania, Africa; 4 College of Pharmacy & Pharmaceutical Sciences, Florida A&M University, Tallahassee, Florida, USA; 5 The Prostate Net, Newark, New Jersey, USA

## Abstract

Although there is significant evidence of a cancer epidemic in Africa, there is limited awareness about cancer in most African countries. By partnering with international organizations and institutions such as the University of Florida and the Prostate Net, the African Organisation for Research and Training in Cancer (AORTIC) is committed to improving cancer advocacy in Africa. This paper presents some of the recent efforts on cancer advocacy in Africa, including the results of a SWOT analysis conducted for the cancer advocacy workshop and the guidelines developed by cancer advocates on best practices for cancer advocacy in Africa. One of the outcomes of these efforts is the ***African Cancer Advocates Consortium (ACAC)*** founded by cancer advocates in Africa to, **“*Make Cancer a Top Priority in Africa*”**. While we have started the work to strengthen cancer advocacy in Africa, we still have a long way to go. Our goal of making cancer a priority in Africa can mainly be achieved by: (1) increasing the manpower for cancer advocacy through education and training; and (2) strengthening the network of cancer advocates across the continent.

## Background

An organization that has been in the forefront of fighting cancer in Africa is the African Organisation for Research and Training in Cancer (AORTIC). AORTIC is committed to improving cancer advocacy in Africa by advancing effective cancer control policies and program implementation in the continent. In line with this goal, AORTIC partnered with the University of Florida and the Prostate Net to implement the first biennial *International Workshop on Cancer Advocacy for African Countries (CAAC)* on November 29, 2011 during the AORTIC’s 8^th^ International Cancer Conference in Cairo, Egypt. To better understand cancer advocacy in Africa, a cancer advocacy SWOT analysis was conducted prior to the cancer advocacy workshop. A SWOT Analysis is a strategic planning tool used to evaluate the strengths, weaknesses, opportunities, and threats involved in a project. We conducted a survey of leading African cancer advocates through online and electronic mail submissions. A copy of the SWOT analysis survey instrument is provided in Additional file 1. The results of the SWOT analysis were used to prepare for the cancer advocacy workshop.

At the end of the CAAC international workshop, the ***African Cancer Advocates Consortium (ACAC)*** was founded to **“*Make Cancer a Top Priority in Africa*”**. The ACAC currently comprises 51 members from diverse countries in Africa and is led by African cancer advocates. The consortium focuses on six advocacy areas:[1]

**1. Political Advocacy**, to lobby in order to impact public policy at local, state, and federal levels;

**2. Education Advocacy** to enhance cancer information and education;

**3. Research Advocacy** to foster high quality cancer research that meets the needs of patients and the community;

**4. Fundraising Advocacy** to raise funds to support cancer research, services, education, and community outreach;

**5. Support Advocacy** to support cancer patients, families, and caregivers; and

**6. Community Outreach Advocacy** to engage and reach out to the community to foster cancer control.

**Figure 1:**
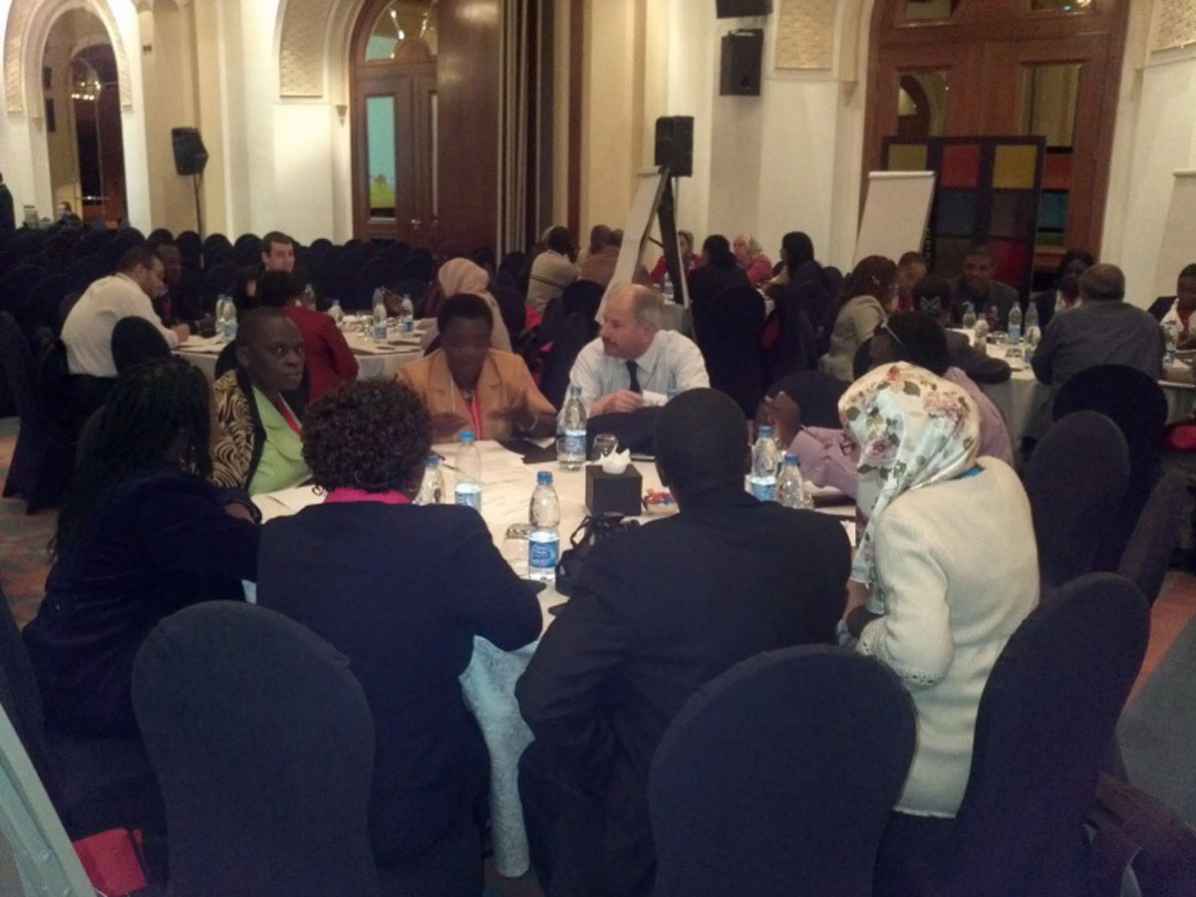
Roundtable discussions by workshop participants to develop best practices for cancer advocacy. The discussions focused on: [1] Education advocacy; [2] Community Outreach advocacy; [3] Political advocacy; [4] Research advocacy; or [5] Fundraising advocacy.

**Figure 2:**
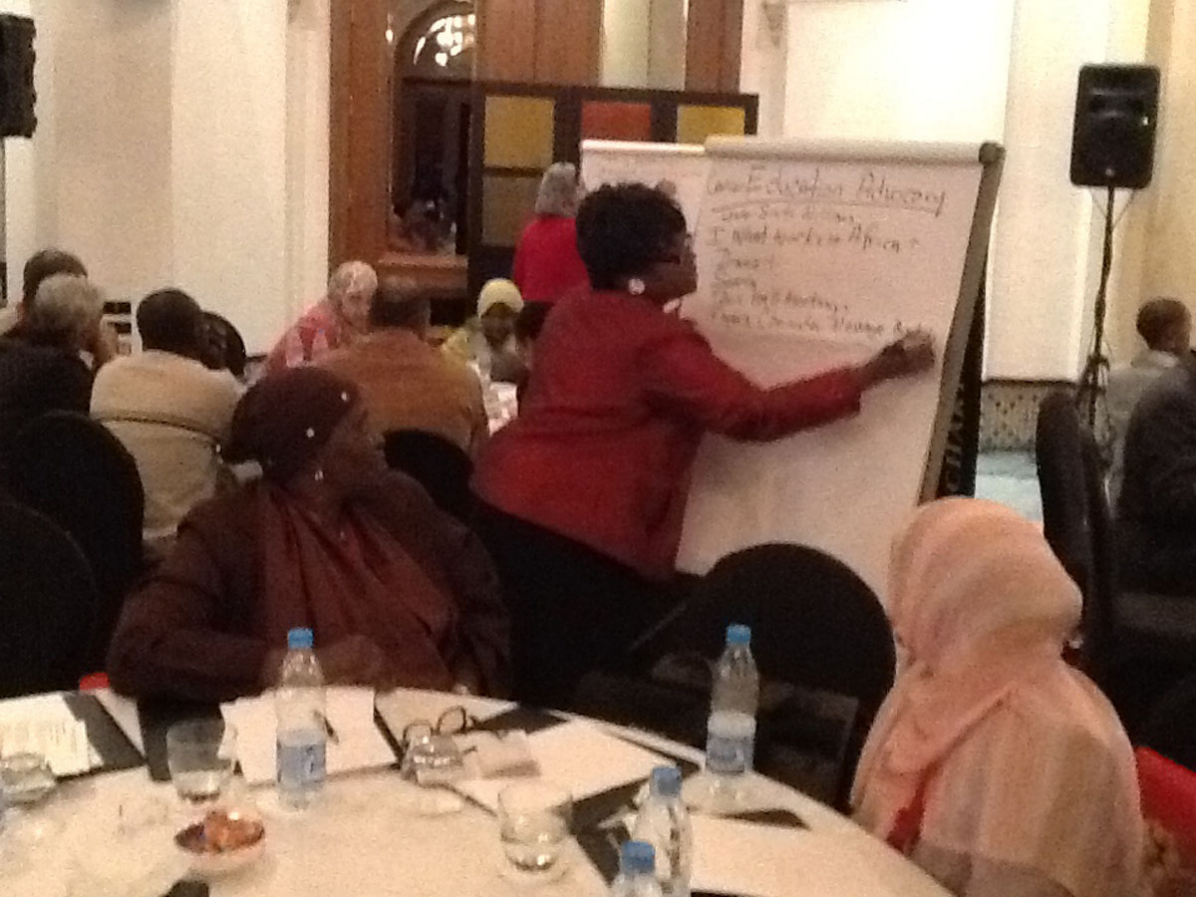
Each discussion group had a group facilitator and Note-taker. The note-taking for Cancer Education Advocacy was conducted by Dr. R. Renee Reams (Florida A&M University, USA).

**Figure 3:**
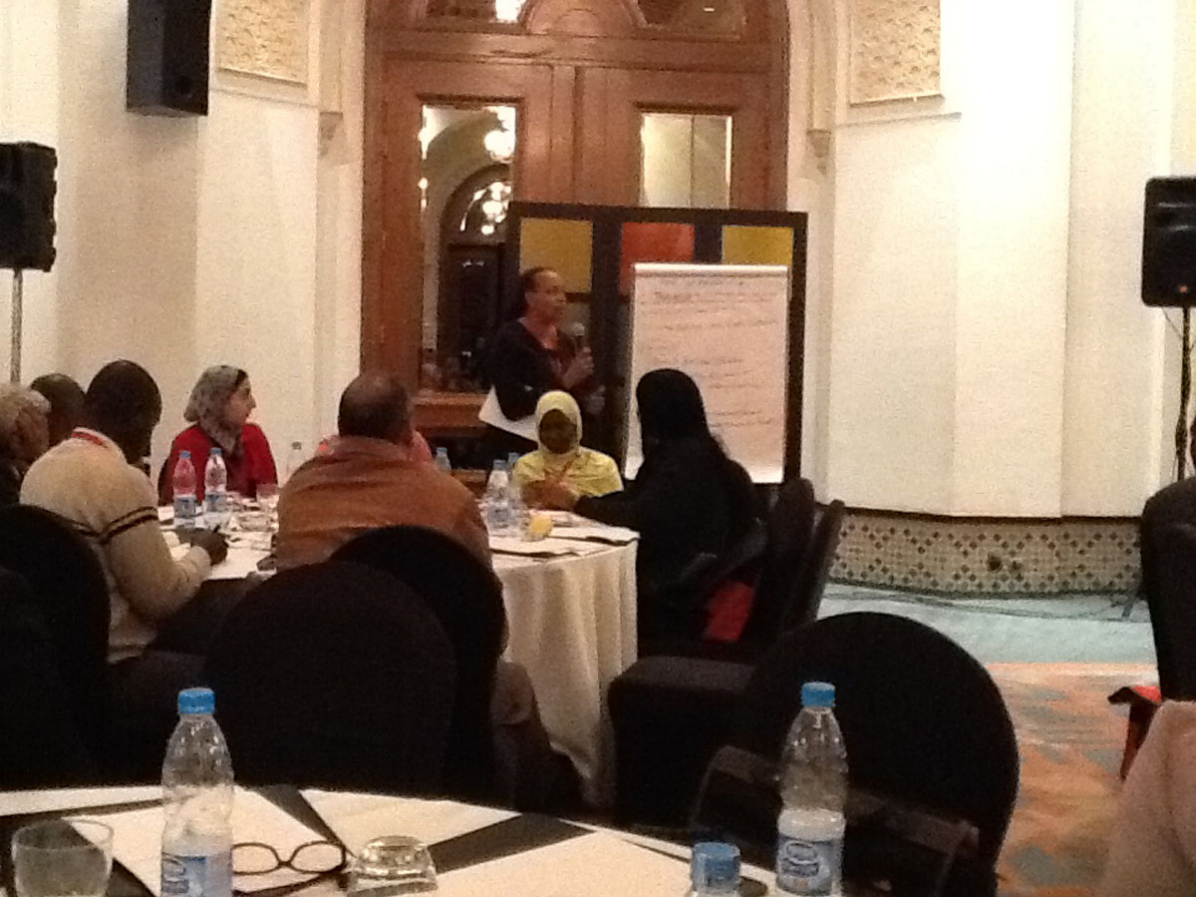
Mary J Scroggins of *In My Sister’s Care* and *The Pathways Project* reporting back on best practices for Research Advocacy.

**Figure 4:**
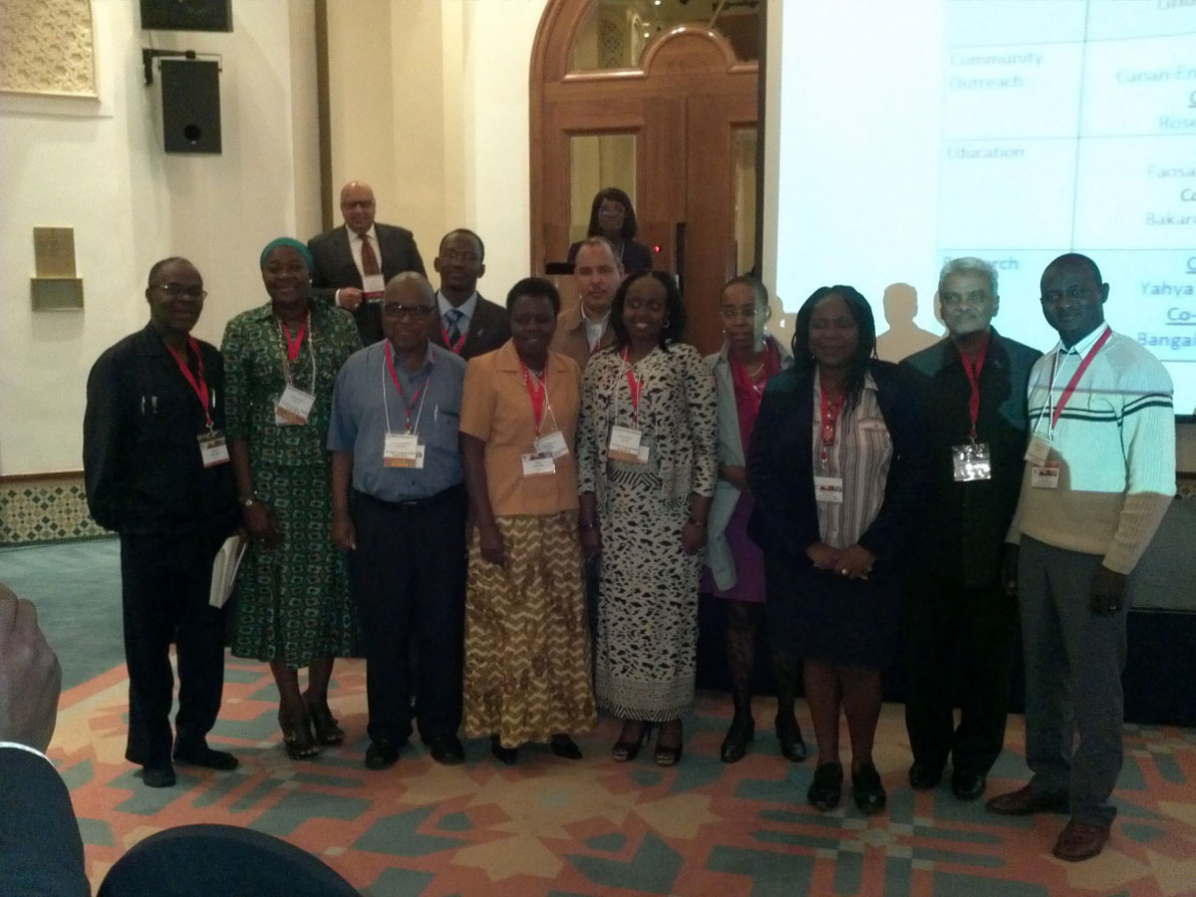
Elected 2011-2013 Leadership Team members for the African Cancer Advocates Consortium (ACAC)

A unique initiative that was implemented during the 2011 workshop session was the development of best practices for cancer advocacy in Africa. The 73 delegates at the workshop self-selected themselves into six groups to develop best practices for [1] political advocacy, [2] support advocacy, [3] fundraising advocacy, [4] community outreach advocacy, [5] education advocacy, and [6] research advocacy. Each group had a facilitator to lead and moderate the discussions, and a recorder to summarize the discussions. Figures 1 to 4 provides a pictorial summary of the best practices discussion.

In this paper, we present the results of the SWOT analysis for the cancer advocacy workshop and the guidelines developed by 73 cancer advocates on best practices for cancer advocacy in Africa.

## Cancer advocacy in Africa: strengths, weaknesses, opportunities and threats

An expert panel of five African advocates participated in the Cancer Advocacy SWOT analyses, identifying the strengths, weaknesses, opportunities and threats linked to cancer advocacy in Africa. The key internal conditions helpful in establishing cancer advocacy in Africa were:

1. Strong women organizations.

2. Strong youth organizations.

3. Widespread use of mobile phone devices and social media that can be used to share cancer information.

4. Willingness of community to participate in health promotion.

5. Willingness of faith-based organization to get involved in cancer advocacy.

6. Willingness of cancer clinicians and scientists to collaborate with advocates.

7. Committed cancer advocates.

8. Funding from private foundations and pharmaceutical companies.

9. Empowered patients who become advocates.

On the other hand, the participants identified some internal conditions harmful to the success of cancer advocacy in Africa. The factors identified by participants were:

1. Competing priorities, such as poverty and illiteracy.

2. Competing public health priorities.

3. Financial barriers.

4. Limited healthcare resources.

5. Lack of awareness and knowledge about cancer advocacy.

6. Lack of knowledge about the disease.

7. Cultural beliefs, including myths and superstitions.

8. Lack of cancer registries to provide accurate and complete cancer data for advocacy.

9. Absence of National Cancer Plans to guide and direct cancer control planning.

10. Lack of collaboration among cancer advocates and non-governmental organizations (NGOs) leading to fragmentation and duplication of cancer advocacy.

11. Poor health care systems.

12. Limited human resources.

While an understanding of the internal conditions that affect advocacy is important, it is also imperative to understand external conditions outside Africa that impacts advocacy in Africa. The helpful external conditions identified by participants were:

1. Funding support for advocacy.

2. Technical support from international organizations.

3. Training opportunities for cancer advocates.

4. Opportunity to join international advocacy networks for support.

5. Collaboration opportunities with clinicians, scientists, and the community.

6. Opportunities to share African realities with advocates from other countries, for example through AORTIC surveys and workshops.

The harmful external conditions identified by participants include:

1. Unnecessary emphasis on malignancies that are not of any burden to the health system in Africa.

2. Emphasis on non-Afrocentric ways of doing things.

3. Competing priorities perceived by international organizations, for example focus on infectious diseases.

4. Lack of funding to support efforts.

5. Misunderstanding of African realities.

Cancer advocacy is still at its infancy in Africa and faces multiple challenges. The top 10 challenges that African advocates face include:

1. Lack of funds.

2. Fragmentation or silo approaches in public healthcare policy framing & implementation

3. Lack of interest by the target population due to the failure of meeting basic needs.

4. Limitations in clinical resources to support advocacy efforts.

5. Competing life/health priorities.

6. Lack of equality in the health care system.

7. Lack of cancer registries.

8. Lack of National Cancer Plans.

9. Lack of political will in dealing with cancer in Africa.

10. Stigmatization of cancer and under-appreciation of the burden of the disease.

The key to successful advocacy in Africa depends on overcoming these challenges. Some of the solutions proposed by participants were:

1. Education and training for advocates, including in areas of grant writing and networking.

2. Local, state, national, and global collaborations to share resources.

3. Creating community awareness for bigger acknowledgement of cancer and its treatment and impact.

4. Lobbying for each country to have a cancer registry.

5. Putting pressure on the government to develop a National Policy and Plan for the prevention and control of cancer.

6. Development of a funding model that will enhance collaboration among cancer NGOs rather than competition for scarce resources.

7. Coordination of cancer funding nationally to eliminate the disparities that exist in different regions, especially with respect to access to appropriate treatments, fair distribution of services and more effective service delivery.

8. Control of the high cost of cancer treatment to make treatment more available in state and private sectors.

9. Development of national policies and infrastructure to manage the burden of cancer in Africa.

10. Development of a platform for addressing cancer advocacy in Africa, led by experienced and committed African cancer advocates.

## Best practices for cancer advocacy in Africa

The first biennial AORTIC Cancer Advocacy workshop fostered the development of best practices for cancer advocacy in Africa. The best practices developed for each type of advocacy is described below.

### Cancer education advocacy

Education advocacy focuses on educating and increasing cancer awareness of patients, family, and lay public using diverse media. The best practices suggested for Education Advocacy were based on the identification of: (i) What works in Africa; (ii) What has not worked in Africa; and (iii) What to avoid. Creativity and innovation are important for effective cancer education. The Education Advocacy working group identified educational channels, sources, sites, and strategies that work in Africa. These include: use of drama to educate the public; providing education during sports events, such as soccer, which is popular in Africa; providing education at places of worship; use of social gatherings such as weddings and funerals; and taking advantages of pre-arranged meetings such as town hall meetings. The group also emphasized the importance of media, such as radio and TV, as well as the new age social media of Face book and Twitter. On the other hand, the use of traditional channels such as fliers and community message boards are also important, especially in rural communities. When it comes to the source of the information, cancer survivors, chiefs, religious leaders and women’s groups were identified as important sources. What has not worked very well in Africa is securing the support of politicians, which makes Political Advocacy very important in Africa. Finally, the group noted that cancer screening must be avoided if there is no funding to support patient treatment and follow-up care if diagnosed with cancer. Screening without a plan for medical care is a big problem and adds to the stigma of cancer in Africa. This is because when patients are diagnosed with cancer and do not have funding to support appropriate care, they are more likely to die. This continues to confirm the fear of most people that cancer automatically means dying. It is thus important to have an effective fundraising effort to support the treatment of those diagnosed with cancer.

### Support advocacy

One of the most important aspects of advocacy is providing support for newly diagnosed cancer patients, survivors, families, friends and caregivers. The AORTIC Support Advocacy working group defined cancer support as ***connecting patients, families, and caregivers for help, hope, and inspiration throughout cancer management and needs***. The best practice identified by the group is that the patient’s voice in expressing his or her needs is essential for Support Advocacy. The support could be in terms of physical, emotional, family, services, information, healthy lifestyle and financial support. Additional best practices are to (i) avoid marginalization of the care of patients; (ii) eliminate disparity of services for underserved patients; (iii) make deliberate multi-sectoral action plans to foster cancer care support in Africa; and (iv) facilitate local and global collaborations for successful Support Advocacy.

### Fundraising advocacy

Advocacy requires significant effort and money. Thus, Fundraising Advocacy is important in order to support all the other types of advocacy. The best practices identified by the Fundraising Advocacy working group was based on what has worked in Africa and included: (i) Fundraising efforts tailored and targeted to those who have been affected by cancers (such as families of survivors) and those who benefit financially indirectly or directly from cancer (such as pharmaceutical companies and hospitals); (ii) Joint fundraising efforts by multiple partners to make a larger impact; (iii) Partnering with local companies or organizations who benefit from the targeted community, such as super market stores; (iv) Submitting special requests to the Minister of Health to support cancer initiatives; and (v) Making public records of successes and name of sponsors.

### Research advocacy

The fight against cancer can only be won when scientific research is tailored to the priorities of cancer patients. Research Advocacy ensures that the voices of cancer patients and survivors are heard by researchers. The best practices established by the Research Advocacy working group are: (i) Training and mentorship for African advocates in the area of Research Advocacy; (ii) Continuous assessment of priorities of cancer patients; (iii) Partnership with scientists for active involvement in grant proposals and research; (iv) Fostering representation of diverse cancer patients in biomedical research through active participants’ recruitment and retention; (v) Encouraging multidisciplinary team-based, innovative research; and (vi) Active involvement in human subjects protection by serving on grant review panels and Institutional Review Boards (IRBs). Furthermore, the group identified the following priority areas to advocate for research: free access to scientific literature, effective and efficient IRBs, increase in innovative research and avoidance of duplicative research, and community-based research that incorporates the needs of patients.

### Community outreach advocacy

Community Outreach Advocacy is defined by the working group as an initiative that goes to the grassroots level. It involves a bi-directional dialogue with the targeted community. Two best practices were identified by the Community Outreach Advocacy working group: (i) Conduct needs assessment to appropriately meet the needs of the community; and (ii) Disseminate information back to the community in a timely manner. To function effectively in a community, advocates should not work alone but develop partnership with key stakeholders in the community. Advocates should also avoid imposing their ideas on the community.

### Political advocacy

Political Advocacy is central to making significant impact on the population as it involves lobbying to impact public policy at local, state, national and global level. The best practices identified by the Political Advocacy working group are: (i) Clearly identifying the outcome desired, for example passing a bill to change public health policy; (ii) Promoting a multi-disciplinary advocacy movement; (iii) Lobbing multiple stakeholders; and (iv) Employing an effective spokesperson for political advocacy. For effective lobbying, the targeted person is very important and has to be an influential person who can shape public policy, such as political leaders, spouses of political leaders, religious leaders, and royalties (kings, chiefs). It is also important to be strategic in getting the attention of public policy change agents. Examples of what has worked in Africa are consistent contact with political officials through personal visits, letters, phone calls, and petitions; use of data and information unique to the constituency of the targeted person; personal stories of a patient from the constituency of the targeted person; and providing opportunity for positive public relations.

## Conclusion

While we have started the work to strengthen cancer advocacy in Africa, we still have a long way to go. Our objective can mainly be achieved by: (1) increasing the manpower for cancer advocacy through education and training; and (2) strengthening the network of cancer advocates across the continent. Thus, the primary goal of the biennial CAAC international conference is to train cancer advocates who will be empowered to engage their communities, develop, and implement cancer health and survivorship programs. Utilizing an innovative framework for its training activities, the biennial workshops will provide the skills to: (1) mobilize the resources within African countries for health promotion, prevention, and survivorship strategies; (2) partner with key stakeholders to accomplish targeted objectives; (3) raise funds to support advocacy activities; and (4) develop and successfully organize community-centered programs.

The theme for the 2^nd^ biennial CAAC workshop, which will take place in South Africa in 2013 is: *“Cancer Advocacy: Tools for Political, Education, Research, Fundraising, Support and Community Outreach Advocacies”.* Based on the feedback from African cancer advocates, there will be a two-day workshop series: (i) a Master Trainer workshop proposed to train 25 master trainers to conduct country- and regional-based advocacy programs in Africa; and (ii) a general workshop conducted by the 25 master trainers as part of their experiential training. Advocates will have the opportunity to share their advocacy programs through an Advocacy Expo and Posters session. In addition, within-continent collaborations of advocacy programs will be fostered through the networking and the ACAC meeting.

The planned 2013 ACAC workshop, which will be held in conjunction with AORTIC 2013 meeting (http://www.aortic-africa.org/index.php/conferences/), will significantly impact cancer advocacy in Africa by providing a forum for advocates, cancer survivors and community leaders interested in finding solutions to the cancer epidemic in Africa to learn, network and initiate collaborative advocacy projects. This workshop is highly innovative because: (i) It will develop a critical mass of Master Trainers in Africa; (ii) It will bring together diverse participants who will learn from the conference and each other, and who in turn will lead the fight against cancer in Africa; and (iii) There is a significant number of partnering African institutions and organizations. AORTIC is dedicated to developing cancer advocacy capability to eliminate cancer burden in Africa through the workshops and the ACAC. The biennial workshops offer unique benefits that cannot be achieved individually.

## List of abbreviations used

ACAC: African Cancer Advocates Consortium; AORTIC: African Organisation for Research and Training in Cancer; CAAC: Cancer Advocacy for African Countries; IRBs: Institutional Review Boards.

## Competing interests

The authors declare that they have no competing interests.

## Supplementary Material

Additional file 1 AORTIC Advocacy SWOT Analysis Survey Click here for file

## Declarations

The authors declare that funding for publication of the article was funded by an award provided by the National Cancer Institute (Award Number R13CA162899).

This article has been published as part of *Infectious Agents and Cancer* Volume 8 Supplement 1, 2013: Selected articles from The International Workshop on Cancer Advocacy for African Countries (CAAC) 2011. The full contents of the supplement are available online at http://www.infectagentscancer.com/supplements/8/S1 The Supplement Editors declare they have no competing interests.
