# Supplementary material for: The African cancer advocacy consortium: shaping the path for advocacy in Africa
Source: Infect Agent Cancer. 2013 Jul 15;8(Suppl 1):S8. doi: 10.1186/1750-9378-8-S1-S8 (PMC3716719; doi:10.1186/1750-9378-8-S1-S8)
Supplement: Additional file 1 — AORTIC Advocacy SWOT Analysis Survey [file 1750-9378-8-S1-S8-S1.pdf]

Appendix I

AORTIC Advocacy SWOT Analysis Survey

Dear Participant,

We are excited to announce the 1st Community Advocacy Workshop at the AORTIC 2011 International Cancer Conference. The primary goal of this workshop is to inform, educate and empower cancer advocates in Africa. To adequately meet the needs of workshop participants, we are conducting a SWOT Analysis for cancer advocacy in Africa. SWOT Analysis is a strategic planning tool used to evaluate the Strengths, Weaknesses, Opportunities, and Threats involved in a project. Once we understand the Strengths and Weaknesses as well as the Opportunities and Threats faced in Africa relative to developing cancer advocacy, the goal is to create an innovative advocacy strategy by answering the following questions:

1. How can we use each strength?
2. How can we stop each weakness?
3. How can we exploit each opportunity?
4. How can we defend against each threat?

This survey should take about 15 - 20 minutes to complete. Please answer the questions candidly. There are no right or wrong answers to the questions asked in this survey. We are interested in your honest opinion only and you may be assured of complete confidentiality. Our goal is to use the information provided to meet the needs of the Community Advocacy Workshop participants. Your name will never be placed on the reports generated from this SWOT Analysis, nor will your responses be linked to you personally during analyses. Please return the completed survey to spressey@cop.ufl.edu or fax to 1- 727-394-6014.

If you have any question(s) or comment(s) about the survey, please contact Prof. Folakemi Odedina at fodedina@cop.ufl.edu / folakemi.odedina@urology.ufl.edu . Thank you for your participation. We look forward to meeting you at the 2011 AORTIC conference.

Sincerely,

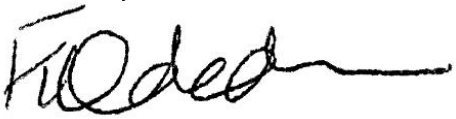

Prof. Folakemi T. Odedina, PhD
Professor, College of Pharmacy
Director, Community Outreach & Minority Affairs
The Prostate Disease Center, College of Medicine
University of Florida
Gainesville, Florida, USA

I. BACKGROUND INFORMATION

Organization Name	

Your Name	

Your Title	

Your Phone #	

Your Email address	

How long have you been an advocate?	☐Within past year (anytime less than 12 months ago)<br>☐Within past 2 years (1 year but less than 2 years ago)<br>☐Within past 5 years (2 years but less than 5 years ago)<br>☐5 or more years ago

What is the average age of the population you serve?	☐Between 30 and 39 years<br>☐Between 50 and 59 years<br>☐Between 70 and 79 years	☐Between 40 and 49 years<br>☐Between 60 and 69 years<br>☐80 years or above

What is the primary gender that you target?	☐Male only<br>☐Both male and female	☐Female only

II. INFORMATION ABOUT THE CONFERENCE

Do you plan to attend the 2011 AORTIC conference in Cairo?	☐Yes ☐No ☐Not sure

Do you plan to attend the Cancer Advocacy Workshop at the 2011 Aortic International Conference?	☐Yes ☐No ☐Not sure

If you plan to attend the Cancer Advocacy Workshop, would you need a travel award to support your participation?	☐Yes ☐No ☐Not sure

III. SWOT ANALYSIS

Please use the space below to list the ***internal conditions*** in your country that are ***helpful*** to establishing cancer advocacy [Strengths].

1.
2.
3.
4.
5.
6.
7.

Please return survey to spressey@cop.ufl.edu .
This survey is supported by the University of Florida, Gainesville, Florida, USA

Please use the space below to list the ***internal conditions*** in your country that are ***harmful*** to establishing cancer advocacy [Weaknesses].

1.
2.
3.
4.
5.
6.
7.

Please use the space below to list the ***external conditions*** outside your country that are ***helpful*** to establishing cancer advocacy [Opportunities].

1.
2.
3.
4.
5.
6.
7.

Please return survey to spressey@cop.ufl.edu .
This survey is supported by the University of Florida, Gainesville, Florida, USA

Please use the space below to list the ***external conditions*** outside your country that are ***harmful*** to establishing cancer advocacy [Threats].

1.
2.
3.
4.
5.
6.
7.

What aspects of community advocacy do you consider is important for your country?

1.
2.
3.
4.
5.
6.
7.

Please return survey to spressey@cop.ufl.edu .
This survey is supported by the University of Florida, Gainesville, Florida, USA

What are the top three challenges you face as an advocate in your country?

1.
2.
3.

How would you propose to overcome the challenges identified above?

Please state any other comments you have below:

Thank you for your participation.

Please return survey to spressey@cop.ufl.edu .
This survey is supported by the University of Florida, Gainesville, Florida, USA
